# Analysis of cytokine profile and growth factors in platelet-rich plasma obtained by open systems and commercial columns

**DOI:** 10.1590/S1679-45082016AO3548

**Published:** 2016

**Authors:** Alberto de Castro Pochini, Eliane Antonioli, Daniella Zanetti Bucci, Luiz Roberto Sardinha, Carlos Vicente Andreoli, Mario Ferretti, Benno Ejnisman, Anna Carla Goldberg, Moisés Cohen

**Affiliations:** 1 Escola Paulista de Medicina, Universidade Federal de São Paulo, São Paulo, SP, Brazil.; 2 Hospital Israelita Albert Einstein, São Paulo, SP, Brazil.

**Keywords:** Platelet-rich plasma, Cytokines, Intercellular signaling peptides and proteins, Centrifugation/methods

## Abstract

**Objective:**

To evaluate growth factors and cytokines in samples of platelet-rich plasma obtained by three different centrifugation methods.

**Methods:**

Peripheral blood of six individuals with no hematological diseases, aged 18 to 68 years, was drawn to obtain platelet-rich plasma, using the open method and commercial columns by Medtronic and Biomet. The products obtained with the different types of centrifugation were submitted to laboratory analysis, including pro-inflammatory cytokines and chemokines by flow cytometry assays, the concentration of fibroblast growth factors-2 (FGF-2) and transforming growth factor-beta1 (TGF-β1).

**Results:**

The diverse separation methods generated systematically different profiles regarding number of platelets and leukocytes. The Medtronic system yielded a product with the highest concentration of platelets, and the open method, with the lowest concentration of platelets. The results of cytokine analysis showed that the different types of centrifugation yielded products with high concentrations of interleukin 8, interleukin 1β. The open system resulted in a product with high levels of interleukin 6. Other cytokines and chemokines measured were similar between systems. The product obtained with the open method showed higher levels of TGF-β1 in relation to other systems and low FGF-2 levels.

**Conclusion:**

The formed elements, growth factors and cytokines in samples of platelet-rich plasma varied according to the centrifugation technique used.

## INTRODUCTION

Platelets are cell fragments derived from megakaryocytes, measuring approximately 2μm in diameter. Their structure contains some organelles, such as mitochondria, microtubules and granules (α, δand λ). The α granules contain more than 30 bioactive proteins, including growth factors, cytokines, adhesion and other molecules, which play a fundamental role in homeostasis and tissue remodeling.^(1,2)^ Platelet proteome analysis reveals that in response to activation by thrombin, human platelets release more than 300 different proteins.^(2)^ These growth factors have a significant impact on the action of mesenchymal cells in the wound healing processes.^(3–6)^ In addition, cytokines and chemokines - which may be present in platelet-rich plasma (PRP) - can assist in the modulation of inflammation and in the resolution of regenerative processes.^(6–10)^ The use of platelets as a rich source of bioactive factors was first suggested as a supplement to allogeneic fibrin glue. However, the availability of growth factors in platelet concentrates aroused great interest in this method for inducing healing and tissue regeneration, which is considered a low-cost alternative,^(11)^ although there has been a gradual change in the cost of the treatment in the last 10 years. A substitute for these platelet concentrates is PRP, a preparation from which leukocytes and erythrocytes are removed, preserving the platelets, which are elements of smaller size and weight.

The use of PRP has been proposed in several health care areas, particularly in orthopedics, sports medicine and dentistry, as a therapeutic adjuvant in tissue regeneration processes. Several investigators associated this type of clinical application of PRP with induction of bone formation or the acceleration of wound healing in tissues, such as ligaments, muscles or tendons.^(4,12–15)^ However, the subject is controversial, since some reports reveal good results with the use of PRP,^(16,17)^ whereas others indicate insufficient or negative results.^(16–18)^

Platelet-rich plasma is a volume of plasma that has a platelet concentration above the reference normal value. The average normal platelet count is 200,000 platelets/μl, but after processing, a concentration of 1,000,000 platelets/μl is expected in PRP, that is, a five-fold enrichment.^(19,20)^ Since PRP is generally developed from autologous blood, it is considered safe for clinical application, for not reacting with the host nor transmitting diseases.^(3,21)^ However, an uncontrolled variable is added to the proposed therapy when preparations derived from patients with immune and inflammatory diseases are used. There are few studies that consistently document the effects of PRP application in patients or even the composition of the preparations.^(22)^ There is no clearer information available on treatment efficiency, duration of effects, incidence and type of adverse events. Moreover, there is no consensus on the optimal composition and the profile of the components present in the various PRP formulations and preparation methods.^(20,22,23)^ Furthermore, several studies showing positive effects of PRP application actually included the use of autogenous bone, in addition to PRP, in the regeneration of skin and bone tissues.^(15)^ One of the outstanding issues is the presence of red blood cells and white blood cells contaminating the PRP, and their role in the success or failure of treatment. Some studies show that the red blood cells present in intraarticular injections can lead to irritation of the synovial membrane, whereas leukocytes may be associated with both tissue protection and greater inflammation.^(4)^ In fact, the presence of leukocytes appears to significantly increase inflammatory cytokines, changing the regenerative potential of PRP,^(20,24)^ inducing pain and functional limitation. On the other hand, other studies show a possible antibacterial function of PRP.

There are several methods to obtain PRP from whole blood.^(21,22)^ The open method, still considered as one of the most efficient, uses centrifugation with subsequent recovery of isolated plasma.^(18)^ There are also columns for obtaining PRP, which are sold as kits with no specifications of their contents. In this study, we compared the PRP obtained by the open method with two commercial products, specifically the Magellan columns (Arteriocyte Medical System, Cleveland, Ohio, USA) and GPSIII (Biomet, Warsaw, Indiana, USA). We analyzed the blood samples before processing, and the PRP obtained after processing, as to the presence and count of formed elements, particularly the platelet count, concentration of transforming growth factor-beta1 (TGF-β1) and fibroblast growth factor-2 (FGF-2), TGF-β1 and basic FGF-2. In addition, we measured the cytokines and chemokines involved in the healing process or with immunomodulatory potential, namely interleukin (IL) 1β, IL-6, IL-8, IL-10, tumor necrosis factor alpha (TNF-α), monocyte chemotactic protein-1 (MCP-1), macrophage inflammatory protein-1 (MIP-1) and regulated on activation, normal T cell expressed and secreted (RANTES). The samples were obtained from patients undergoing surgery for injuries, with the rationale to assess whether there were or not pro-and anti-inflammatory factors present, and whether the PRP samples were free or not from formed elements possibly harmful to treatment.

## OBJECTIVE

To evaluate the presence of growth factors and cytokines in samples of platelet-rich plasma, obtained by three different centrifugation methods.

## METHODS

### Obtaining platelet-rich plasma

Peripheral blood was drawn from six individuals undergoing surgery for rotator cuff injury, with no blood disease, aged 18 to 68 years, to obtain PRP by applying the open method and, in parallel, by using the Magellan and GPSIII columns. The first method is a platelet separation column used in a single centrifugation step, with no indication of contamination with erythrocytes, but with the information that leukocytes are present in the concentrate. The second column is used in a two-step protocol, in which PRP is recovered from the intermediate layer between the red cells and the supernatant. Volumes of 26mL and 60mL of peripheral blood were collected, respectively, for each kit. The samples were processed by the local representative of each brand, according to the protocol established by the manufacturers. These two brands have in common the fact that both prepare a PRP richer in leukocytes and platelets.^(22)^

For the analysis of the open system to obtain PRP, developed by Anitua et al.,^(21)^ samples of 20mL of peripheral blood were collected with a 19 gauge needle into tubes containing sodium citrate, from four subjects aged from 54 to 63 years. The material was centrifuged at 650g for 8 minutes, at room temperature, and the plasma supernatant was collected from the third fraction, next to the leukocytes and erythrocytes, a fraction rich in platelets and bioactive factors, but trying to avoid collection of leukocytes.^(25)^

Afterwards, within a maximum period of one hour after collecting the PRP samples by three different methods, the samples were evaluated in a clinical laboratory, with no thrombin activation or treatment with calcium chloride to induce formation of fibrin. The remaining samples were stored at −20°C until analysis.

The study was conducted in full compliance with the guidelines of the Research Ethics Committee of the *Hospital Israelita Albert Einstein*, under protocol 07/733, CAAE: 0182.0.028.000-07. The samples were collected after obtaining written Informed Consent from all donors.

### Laboratory analysis of the components of the platelet-rich plasma

The following parameters were measured in the PRP samples: concentration of platelets, leukocytes and erythrocytes, mean platelet volume (MPV), hemoglobin and hematocrit, in the clinical laboratory of the *Hospital Israelita Albert Einstein*.

### Analysis of growth factors

The growth factors FGF-2 (basic) and TGF-β1 were measured using Quantikine Human FGF basic Immunoassay kit (R&D) and Human TGF-β1 ELISA Set, (BD OptEIA™), respectively, following the manufacturer instructions. A volume of 100μL of each sample was incubated with RD1-43 reagent or Ab capture buffer for 2 hours (after activation with 1N HCl, at a dilution of 1:25 for 60 minutes at 4°C, followed by neutralization with 1N NaOH 1:25) at room temperature. After washing, the samples were incubated with conjugated FGF-2 or TGF-β1 substrate for 30 minutes. The reaction was stopped by adding the stop solution and absorbance readings were obtained in a microplate reader (DTX 880 Multimode Detector -Beckman Coulter, Inc.) at a wavelength of 450nm for FGF-2, and 570nm for TGF-β1.

### Analysis of inflammatory cytokines

The analysis of inflammatory cytokines and chemokines were performed using the Cytometric Bead Array (CBA) method, with Human Inflammatory Cytokines (IL-8, IL-1β, IL-6, IL-10, TNF, IL-12p70) and Human Chemokine (IL-8, RANTES, MIG, MCP-1, IP-10) kits, both from BD Pharmingen (San Diego, CA), following the manufacturer instructions. In short, a serial dilution curve was prepared using a standard sample of cytokines. For the reaction, volumes of 50μL of each sample were incubated with a specific mixture of beads for each of the analytes, followed by a reaction with the secondary antibody conjugated with PE Detection reagent, for one hour, at room temperature. Then the samples were washed and resuspended with wash buffer, and analyzed by flow cytometry (FACSAria, BD Biosciences, San Jose, CA). Data were analyzed using the FlowJo (TreeStar, Ashland, OR) and the BD CBA softwares.

### Statistical analysis

Mean and standard error were calculated for all variables, using the GraphPad Prism software version 6 (GraphPad Software, Inc., La Jolla, CA). The analysis of variance (ANOVA) for nonparametric data was used to assess the variables. The p values <0.05 were defined as level of significance.

## RESULTS

The profile and composition of the platelet concentrate obtained by the three methods are displayed in table 1. Significant differences were observed in the PRP composition of the six samples submitted to different methods. Despite the variability among the samples, the two columns generated systematically different profiles for each sample with regard to the number of platelets and leukocytes. The highest concentration of platelets was obtained using the Magellan column, on average 2.7 (2.11 to 3.95) times larger than GPSIII. The data indicated a concentration of only 1.5 times obtained with GPSIII column, whereas the Magellan column was able to concentrate the platelets about 5 times. However, there was no difference in the mean platelet volume in any samples, indicating an absence of platelet lysis, a factor that may explain the lower values.

**Table 1:** Profile and composition of the platelet concentrate

System	Platelets (x 10^3^/μL)	Mean volume (mL)	Leukocytes (x 10^3^/μL)	Hematocrit (%)	Hemoglobin (g/dL)
GPS III	606.33±84.7	6.7±1.3	25.03±1.9	14.23±5.1	5.65±1.4
Magellan	1630.25±271	6.4±1.3	12.96±1.87	6.96±1.4	3.1±0.6
Open	55±8.6	5.65±0.27	0.6±0.2	0.025±0	0.13±0.1

Reference values for platelets: from 150 to 450 × 10^3^/μL; for mean volume: from 6.5 to 11mL; (n=6).

### Analysis of TGF-β1 and FGF-2 growth factors

Despite the low number of platelets in the PRP prepared by the open method, the resulting product contained the desired bioactive molecules. In fact, the TGF-β1 concentration observed was the largest of the three preparations.

**Figure 1:**
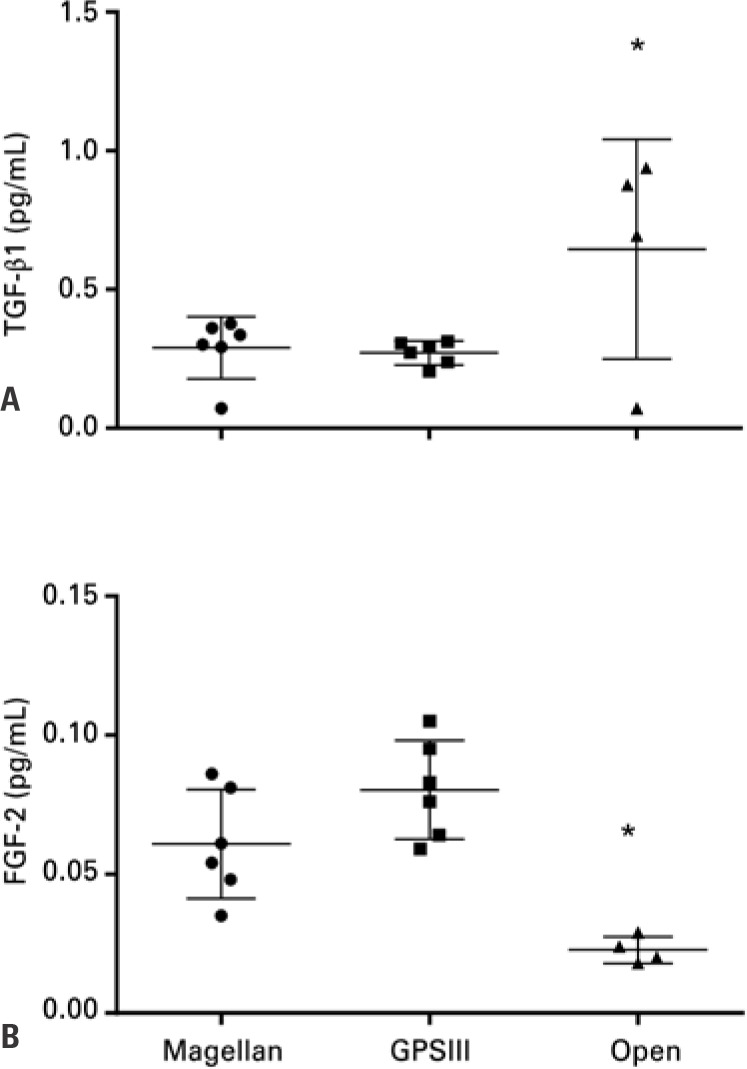
Growth factors concentration. (A) transforming growth factor beta 1 and (B) fibroblast growth factor 2 analyzed in platelet-rich plasma obtained by Magellan (n=6), GPSIII (n=6), and open (n=4) methods *Comparison of open system versus GPSIII, p <0.05. TGF-β1: transforming growth factor-beta1; FGF-2:fibroblast growth factors-2.

The presence of FGF-2 (Figure 1A) also showed high variability, but mostly inverted, in relation to TGF-β1 (Figure 1B).

### Cytokines and chemokines

**Figure 2:**
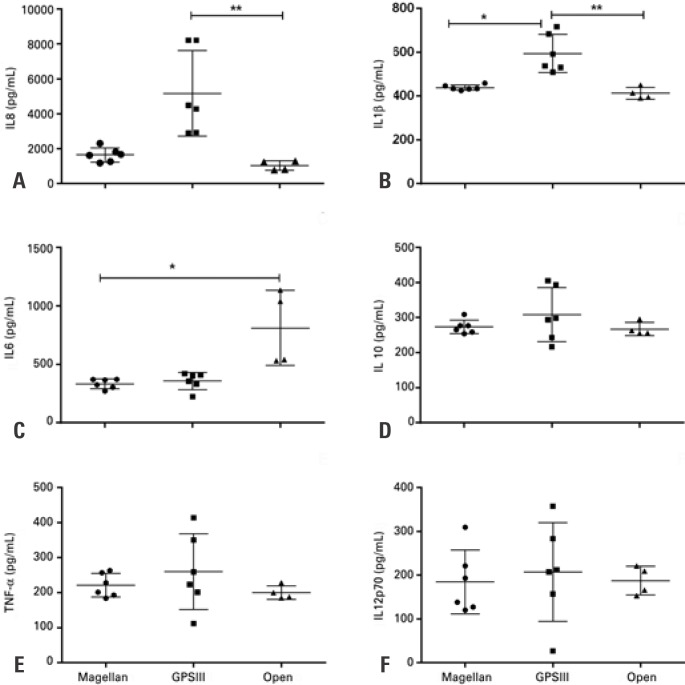
Interleukin levels in platelet-rich plasma measured by Magellan (n=6), GPSIII (n=6), and open (n=4) methods. (A) IL-8; (B) IL-1β; (C) IL-6; (D) IL-10; (E) tumor necrosis factor (TNF-α); (F) IL-12p70 * p value <0.05; ** p value <0.01. TNF-α: tumor necrosis factor alpha; IL: Interleukin levels.

**Figure 3:**
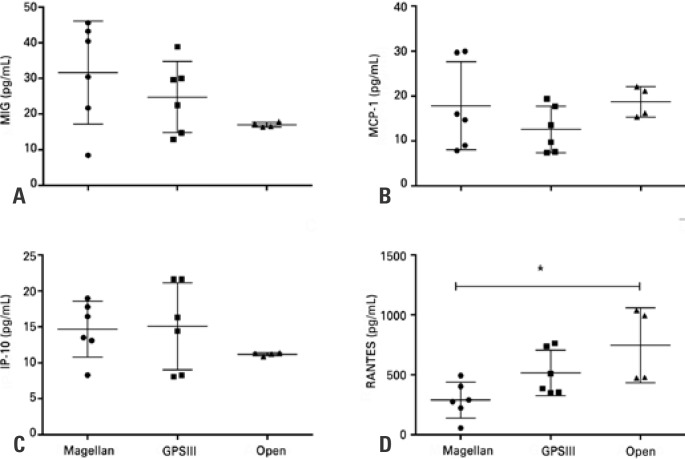
Chemokine levels measured in platelet-rich plasma samples by Magellan (n=6), GPSIII (n=6), and open (n=4) methods * p value <0.05. MIG: monokine induced by gamma; MCP: monocyte chemotactic protein; IP: inducible protein; RANTES: regulated on activation, normal T cell expressed and secreted.

The results of cytokine and chemokine analyses are shown in figures 2 and 3, respectively. The amounts of cytokines were similar in the samples obtained by the three methods. In the samples obtained with GPSIII, this rate was even higher when compared to the Magellan samples (Figure 2A). The same occurred in relation to IL-1β, because the samples obtained with GPSIII showed a significantly greater amount than the other groups (Figure 2B). On the other hand, although the platelets have not been recovered with the open method, the IL-6 levels observed were consistently higher in the latter method than in the other two (Figure 2C). The other cytokines, IL-10, TNF-α and IL-12p70 (Figures 2D, 2E and 2F), were similar in the three types of samples.

The results show the presence, albeit moderate, of MIG, MCP-1, and IP-10 chemokines in the concentrates obtained by the three methods (Figure 3A, 3B and 3C), unlike RANTES, in which they were present in larger amounts than expected, especially in the samples obtained by the open system (Figure 3D).

## DISCUSSION

The results of this study show a wide variation, depending on the type of preparation, even in the same collected sample. One wonders, after all, what is the desired composition of a PRP? Above all, what are the unwanted components that can cause side effects and tissue damage when they are used? It has been described that the final content of the PRP obtained from commercial methods has a higher concentration of red blood cells, which apparently does not induce inflammatory complications due to some tissue reaction to red blood cells.^(26,27)^ However, in this study the application in orthodontic treatment was tested, thus enabling different inflammatory effects according to the tissue assessed. In fact, in the case of skeletal muscle repair, it is recommendable to avoid contamination with red blood cells. The iron contained in the heme molecules catalyzes the formation of free radicals, which, in turn, can induce not only bacterial death, but also apoptosis of tissue cells in response to pro-inflammatory signaling.^(28)^ The number of erythrocytes and leukocytes present in the GPSIII preparation was almost twice as that found in the Magellan column preparation. The latter, in contrast, produced samples with a platelet concentration three times greater, in the recommended range, when compared to other methods.

On the other hand, the mean platelet volume remained proportional in all methods, indicating that the final concentration obtained is not an artifact due to platelet lysis during the centrifugation process. It was clear that a different final product is obtained in each method. We concluded, therefore, that the differences in the number of platelets observed are due to the composition of the columns, or to the procedures indicated by the manufacturer. The open method resulted in a preparation with the lowest concentration of platelets, which was an unexpected result. During the open process, there appears to be a loss of platelets, but it was impossible to specify the reason, since the loss was significant. In the accepted definition of PRP, it should contain at least 200,000 platelets/μL.^(28)^ Nevertheless, in the samples obtained by the open system, the other analyzes were performed to assess whether these preparations, despite the low concentration of platelets, contained or not the factors considered important for the desired therapeutic effect. It is important to note that the product obtained with the GPSIII column showed a significantly higher number of leukocytes compared to the Magellan column, and the same occurred with the hematocrit. There was also high levels of hemoglobin in the PRP samples prepared with two commercial columns (Magellan and GPSIII).^(26,29)^

In contrast, the measurement of TGF-β1 and FGF-2 growth factors levels showed an increased TGF-β1 level only in the preparation obtained using the open system, indicating that the concentration of TGF-β1 and FGF-2 growth factors in the PRP it is not necessarily related to the mass of the recovered platelets. This result contrasts with previous studies that suggest a direct relation between the concentration of platelets and other formed elements in the preparations, and TGF-β1 and FGF-2 levels.^(3,24)^ The higher concentration of TGF-β1 found in the PRP obtained with the open system may have been due to the circulating TGF-β1 in plasma. On the other hand, it is well-known that obtaining reproducible measurements of TGF-β1 is very difficult.^(30)^ Some studies indicate serum levels in the range of 0.1 to 25ng/mL^(29,30)^ or approximately 5ng/mL.^(31)^ In comparison, the values obtained in our study are much lower. According to Veselý et al.,^(31)^ FGF-2 is normally undetectable in serum, with very low values, as we observed. However, although they are classified as generators of leukocyte-rich PRP,^(22)^ thus having the potential to produce larger quantities of TGF-β1, only the GPS III preparation reached statistically significant levels compared to the open system. The wide variation of data, even in triplicate simultaneous analyses, indicates that the variation in these values should not have great biological significance. In fact, the normal serum levels of TGF-β1 reported in the literature range from 4.83 to 55.05ng/mL,^(31)^ confirming that all the values of TGF-β1 obtained are, in fact, lower than the expected. The values measured in this study are equivalent to those obtained by other authors^(29)^ and probably correspond to the platelet TGF-β1, because the plasma samples were subjected to an activation process to release TGF-β1 dimerized with latency-associated peptide (LAP).^(30)^

In parallel, our results show that obtaining PRP by the three methods described produces considerable amounts of IL-6, IL-1β, IL-8 and TNF-α. All PRP samples showed high IL-8 levels as compared to the reference values reported in literature, that is, 1.2 to 1.5ng/L.^(24,26)^ Fibroblast proliferation, with matrix production and fibrous scarring, is a major characteristic of the final stages of healing, and various growth factors have been studied with a possible role in improving the healing of target tissues.^(32)^ The fibroblasts proliferate in response to stimulation by cytokines and factors, such as IL-1, IL-6, IL-4, TNF-α, FGF-2 and TGF-β1– precisely the factors released by the platelets and, for this reason, measured in this study. These results suggest that the use of PRP in cartilaginous tissue can cause a strong pro-inflammatory effect. In fact, it was shown that IL-6 is associated with pro-inflammatory mechanisms in cartilage and, with IL-1 and TNF-α, participates in the cartilage degradation mechanism.^(33)^ Moreover, IL-6 and TNF-α are also associated with the healing of muscle injury,^(34)^ and TGF-β1, through IL-6, participates in transdifferentiation of fibroblasts into myofibroblasts.^(35)^ However, it is important to note that, in this study, the TGF-β1 levels did not follow the high IL-6 values observed. Finally, IL-10, which is considered an anti-inflammatory cytokine, was also present in concentrations higher than expected.^(36)^ There is consensus that circulating IL-10 values may vary widely and, in particular, they increase in response to inflammatory stimuli.^(36–38)^

These considerations are important, particularly because the samples were obtained from patients with surgical indication for the treatment of rotator cuff injury and, therefore, probably presenting active inflammatory process. Thus, it is possible that individuals treated with anti-inflammatory drugs, patients with other lesions, or even those with no active processes, generate preparations with profiles that are very different from those we observed.

It becomes increasingly important to validate the use of PRP, assessing its application in specific diseases, by selecting the target tissues and controlling the heterogeneity of the patients according to their demographic characteristics. Clinical trials should also include the optimization of the preparation, by choosing the best method, the dosage of components present in the preparations, volume of the concentrate, number of applications, whether or not to include leukocytes, and any other confounding factors that may render the use of PRP controversial.^(39)^ A recent study reviewed the published clinical trials on the clinical use of PRP in the treatment of musculoskeletal injuries, and concluded that there is insufficient evidence for the use of this therapy. According to the authors, the standardization of PRP preparation methods is one of the critical points that prevents efficacy of clinical use of PRP from being confirmed.^(40)^

Our results corroborate the literature, demonstrating that diverse PRP preparation methodologies generate products with completely different compositions, both in regard with formed elements and cytokines and growth factors, which probably result in totally different therapeutic effects, hindering the clinical assessment of PRP.

## CONCLUSION

To date, the clinical use of platelet-rich plasma has not yet presented reliable evidence and proven efficacy, in part due to the absence of clear methodology standards for obtaining and characterizing it. The concentration of formed elements, growth factors and cytokines in samples of platelet-rich plasma varied according to the centrifugation method utilized. The method using the Magellan system resulted in a preparation with higher platelet concentration, a main feature of the platelet-rich plasma, but with variability in the concentration of growth factors and cytokines. The validation of the therapeutic use of platelet-rich plasma requires strictly controlled clinical trials, regarding the method for obtaining it and the characteristics of the donors. Knowledge about the composition of the different preparations should contribute to the associations of the use of platelet-rich plasma with the patients' clinical outcomes.
